# Toolbox for studying neurovascular coupling *in vivo*, with a focus on vascular activity and calcium dynamics in astrocytes

**DOI:** 10.1117/1.NPh.9.2.021909

**Published:** 2022-03-14

**Authors:** Cam Ha T. Tran

**Affiliations:** University of Nevada, Reno School of Medicine, Department of Physiology and Cell Biology, Reno, Nevada, United States

**Keywords:** two-photon imaging, optogenetics, astrocytes, calcium, neurons, three-dimensional volume imaging, endothelial cells, vascular smooth muscle cells, pericytes

## Abstract

**Significance:**

Insights into the cellular activity of each member of the neurovascular unit (NVU) is critical for understanding their contributions to neurovascular coupling (NVC)—one of the key control mechanisms in cerebral blood flow regulation. Advances in imaging and genetic tools have enhanced our ability to observe, manipulate and understand the cellular activity of NVU components, namely neurons, astrocytes, microglia, endothelial cells, vascular smooth muscle cells, and pericytes. However, there are still many unresolved questions. Since astrocytes are considered electrically unexcitable, ${\mathrm{Ca}}^{2+}$ signaling is the main parameter used to monitor their activity. It is therefore imperative to study astrocytic ${\mathrm{Ca}}^{2+}$ dynamics simultaneously with vascular activity using tools appropriate for the question of interest.

**Aim:**

To highlight currently available genetic and imaging tools for studying the NVU—and thus NVC—with a focus on astrocyte ${\mathrm{Ca}}^{2+}$ dynamics and vascular activity, and discuss the utility, technical advantages, and limitations of these tools for elucidating NVC mechanisms.

**Approach:**

We draw attention to some outstanding questions regarding the mechanistic basis of NVC and emphasize the role of astrocytic ${\mathrm{Ca}}^{2+}$ elevations in functional hyperemia. We further discuss commonly used genetic, and optical imaging tools, as well as some newly developed imaging modalities for studying NVC at the cellular level, highlighting their advantages and limitations.

**Results:**

We provide an overview of the current state of NVC research, focusing on the role of astrocytic ${\mathrm{Ca}}^{2+}$ elevations in functional hyperemia; summarize recent advances in genetically engineered ${\mathrm{Ca}}^{2+}$ indicators, fluorescence microscopy techniques for studying NVC; and discuss the unmet challenges for future imaging development.

**Conclusions:**

Advances in imaging techniques together with improvements in genetic tools have significantly contributed to our understanding of NVC. Many pieces of the puzzle have been revealed, but many more remain to be discovered. Ultimately, optimizing NVC research will require a concerted effort to improve imaging techniques, available genetic tools, and analytical software.

## 1 Introduction

Neurovascular coupling (NVC) is an essential control mechanism in the regulation of cerebral blood flow (CBF). This process involves multiple cell types, including neurons, glia (e.g., astrocytes), and vascular cells (e.g., vascular smooth muscle cells), which collectively form the neurovascular unit (NVU). Members of the NVU collectively act in an integrative fashion to respond to increased neuronal activity by increasing local brain blood flow, a process termed functional hyperemia.^1^^,^^2^ Understanding the signaling pathways that contribute to NVC and the regulation of blood flow in the brain so as to match metabolic demands will require insights into the activity of cellular components of the NVU. Advances in imaging tools, such as two-photon laser scanning microscopy (TPLSM) in combination with the development of genetic tools, including genetically encoded calcium indicators (GECIs) and Cre-lox technology, have significantly advanced our understandings of NVC and CBF regulation.^3^^–^^12^

It has been proposed that, under normal physiological conditions, increases in neuronal activity trigger the release of synaptic glutamate, which subsequently activates two different signaling pathways that ultimately elicit functional hyperemia: neuronal-dependent signaling pathways, and astrocyte-dependent signaling pathways.^1^^,^^13^ However, current evidence has raised fresh questions regarding whether one of these signaling pathways takes precedence or both operate concurrently under certain conditions. As such, the sequence of events from increased neuronal activity to vasodilation remains to be validated. Techniques for measuring changes in vascular reactivity and neural ${\mathrm{Ca}}^{2+}$ dynamics are critical for understanding the underlying NVC mechanisms and how they impact blood delivery to meet the moment-to-moment needs of neurons. In this brief review, we highlight some currently available tools for studying the NVU—and thus NVC—with a focus on vascular reactivity and astrocytic ${\mathrm{Ca}}^{2+}$ dynamics *in vivo*. We also discuss the utility and limitations of these tools in ongoing NVC research.

## 2 Neurovascular Coupling

NVC forms the basis of functional brain imaging techniques such as positron emission tomography (PET), single-photon emission computed tomography (SPECT), functional magnetic resonance imaging (fMRI), and functional near-infrared spectroscopy (fNIRS), which continue to play important roles in the study of hemodynamics. But how the signals captured by these techniques relate to neuronal activity remains incompletely understood. Accordingly, a rigorous understanding of the interactions between members of the NVU at micrometer-to-millimeter scales of vascular and neural reactivity is critical for more accurate interpretations of functional imaging data. New imaging techniques, in combination with the development of various transgenic mouse models, have significantly advanced our understanding of NVC at the cellular level.^3^^–^^12^ In addition, the introduction of completely awake *in vivo* multiphoton fluorescence imaging has enhanced our ability to examine the interaction of members of the NVU without the confounding effects of anesthesia.^14^^–^^17^ In this brief review, we focus our discussion primarily, but not exclusively, on data acquired from the rodent barrel cortex in the primary somatosensory cortex, a system that has been widely used to investigate NVC.^18^ Two major working models of NVC are considered: (1) activation of neurons directly triggers signaling pathways that release vasoactive agents and cause vasodilation and (2) activation of neurons elicits functional hyperemia indirectly through astrocytes.

### 2.1 Direct Neuronal Activation of Blood Vessels

Inputs from thalamocortical glutamatergic afferents to the neocortex are critical for initiating NVC and subsequent functional hyperemia.^19^^–^^21^ Synaptic release of glutamate induces neuronal ${\mathrm{Ca}}^{2+}$ elevation through activation of N-methyl-d-aspartate (NMDA) and α-amino-3-hydroxy-5-methyl-4-isoxazole propionic acid (AMPA) receptors, leading to activation of ${\mathrm{Ca}}^{2+}$-dependent enzymes such as neuronal nitric oxide synthase (nNOS) and cyclooxygenase 2 (COX-2), which subsequently generate the vasodilators nitric oxide (NO) and prostanoids, respectively.^1^^,^^20^ Lecrux et al. identified pyramidal neurons as “neurogenic hubs” that release COX-2 products and glutamate, which act directly or indirectly through astrocytes, respectively, to drive sensory-induced vascular responses.^22^ The observed reduction in functional hyperemia in $\mathrm{COX}2^{-/-}$ mice,^23^ and in the presence of blockers of COX-2 synthesis pathways,^6^^,^^24^ taken together with staining showing COX-2 processes in contact with cortical microvessels,^25^ strongly support a role for neuronally released COX-2–dependent prostanoids in NVC. One of the prostanoids proposed to be involved in functional hyperemia is prostaglandin E2 (${\mathrm{PGE}}_{2}$). However, the evidence on this point appears to be conflicting. ${\mathrm{PGE}}_{2}$ directly applied to isolated pressurized parenchymal arterioles in the absence of neuronal influences causes constriction rather than dilation,^26^ a finding consistent with *in vivo* data.^27^ Yet other studies have reported that ${\mathrm{PGE}}_{2}$ dilates cerebral arteries.^28^^,^^29^ The discordance in these data could be attributable to differences in preparations used as well as the age of the animals under study, given that ${\mathrm{PGE}}_{2}$ theoretically could elicit either vasodilation or vasoconstriction depending on which receptors (i.e., ${\mathrm{EP}}_{4}$ or ${\mathrm{EP}}_{1}$) are expressed and activated. Interestingly, a recent study reported that PGE2 indirectly elicited intracerebral arterioles dilation via propagating vasodilatory signals initiated at the capillaries^30^ suggesting the discrepancy could be the vascular target. Similar to the case for COX-2-dependent prostanoids, the gaseous neurotransmitter NO, which is synthesized and released rapidly in response to neuronal activation,^31^ has been reported to contribute to functional hyperemia in rodents^6^^,^^11^^,^^12^^,^^32^^–^^36^ and recently in humans.^37^ However, direct activation of the vasculature by neuronally derived NO may be more complex than initially thought, since (1) mice deficient for type I nNOS do not show significant alterations in functional hyperemia^36^ and (2) NO donors restore the inhibitory effects of nNOS blockade in the cortex^32^ but not in the cerebellum.^12^ Thus, whether NO acts as a mediator^11^^,^^12^ or a modulator^32^ remains to be determined. Complexities in parsing the involvement of COX-dependent pathways and the NO pathway in NVC could reflect the potential of the two signaling pathways to work concomitantly or impede one another.

### 2.2 Astrocytes Relay Neuronal-Activation Status to Blood Vessels

Astrocytes, the most abundant cell type in the central nervous system (CNS), exhibit a tiled distribution, occupying largely nonoverlapping territories such that only the farthest reaches of their processes come into close proximity with each other. Astrocytes interact with multiple cell types in addition to other glial cells, including neurons, and vascular cells, and release numerous substances upon activation, many of which have functions that remain unclear.^38^ Since astrocytes intimately interact with both neurons and vascular cells—the former via fine processes^39^ and the latter via a specialized cellular compartment termed endfeet^40^^,^^41^—they are well-positioned to relay neuronal information to the microvasculature, a structural relationship that underscores the well-recognized role of astrocytes in NVC (for a detailed review, see Refs. 42–44).

Despite this general appreciation, the mechanistic details of NVC, including the contribution of astrocytic ${\mathrm{Ca}}^{2+}$ elevations, remain incompletely understood. It has been posited that synaptic release of glutamate during increased neuronal activity activates metabotropic glutamate receptors on astrocytes, resulting in an increase in astrocytic ${\mathrm{Ca}}^{2+}$ that activates the phospholipase A2-dependent pathway and leads to the production of a series of vasodilatory metabolites, including epoxyeicosatrienoic acids (EETs).^45^ Alternatively, activity-dependent vasodilation has been attributed to the opening of $K_{\mathrm{IR}}$ channels in arteriole smooth muscle cells by perivascular $K^{+}$, released from astrocytes via ${\mathrm{Ca}}^{2+}$-dependent activation of large-conductance ${\mathrm{Ca}}^{2+}$-activated $K^{+}$ (BK) channels expressed in astrocytic endfeet.^46^ Recent work from Thakore et al. suggested the involvement of transient receptor potential ankyrin 1 (TRPA1) channels in brain capillary endothelial cells in initiating NVC.^47^ Evidence from *ex vivo* brain slices has further shown that elevations in astrocytic endfoot ${\mathrm{Ca}}^{2+}$ induced by neuronal afferent stimulation^29^ or uncaging ${\mathrm{Ca}}^{2+}$ using photolysis^48^^,^^49^ are accompanied by delayed vasodilation. The availability of oxygen (and thus lactate), as well as adenosine levels, have been proposed to affect the polarity (contraction versus dilation) of the astrocyte-mediated vascular response.^50^ In addition, normal astrocytic endfoot ${\mathrm{Ca}}^{2+}$ concentrations (∼300  nM) have been reported to induce vasodilation, whereas high ${\mathrm{Ca}}^{2+}$ concentrations (∼700  nM) elicit vasoconstriction.^49^

Evidence from *in vivo* studies, however, has introduced some uncertainties regarding the role of astrocytic ${\mathrm{Ca}}^{2+}$ elevations in initiating functional hyperemia, with some studies reporting a rapid onset of astrocytic ${\mathrm{Ca}}^{2+}$ transients after sensory stimulation^8^^,^^51^ and others reporting astrocytic ${\mathrm{Ca}}^{2+}$ elevations in different astrocytic subcellular compartments that are delayed relative to functional hyperemic responses.^6^^,^^9^ There are also reports of functional hyperemic responses to sensory stimulation in the absence of a rise in astrocytic ${\mathrm{Ca}}^{2+}$^52^ or the presence of only sporadic astrocytic ${\mathrm{Ca}}^{2+}$ events.^9^ Recent work demonstrated that a subpopulation of astrocytic ${\mathrm{Ca}}^{2+}$ signals exhibits temporal dynamics similar to those of neurons.^53^ The authors of this study suggested that the subset of ${\mathrm{Ca}}^{2+}$ signals observed in microdomains within fine processes and endfeet could be the driving force for activating signaling pathways that trigger the release of vasodilators. However, direct evidence for the involvement of these fast, discrete microdomain-localized events in functional hyperemia is currently lacking. Furthermore, the sequence of events leading to astrocytic ${\mathrm{Ca}}^{2+}$ elevations in response to increased neuronal activity is still unclear. In particular, although manipulations of inositol 1,4,5-trisphosphate (${\mathrm{IP}}_{3}$)-dependent ${\mathrm{Ca}}^{2+}$ levels in astrocytes have suggested a role for ${\mathrm{IP}}_{3}$ in NVC, knockout of type 2 ${\mathrm{IP}}_{3}$ receptor (${\mathrm{IP}}_{3}R2$), which eliminates detectable ${\mathrm{Ca}}^{2+}$ signals in these cells, does not appear to alter functional hyperemia.^9^^,^^52^ Interestingly, there are reports that revealed that astrocytes differentially regulate NVC at the capillary and arteriole levels.^54^^,^^55^ Yet most recently, this differential regulation has been challenged.^56^

## 3 Synthetic and Genetically Engineered ${\mathrm{Ca}}^{2+}$ Indicators

${\mathrm{Ca}}^{2+}$ is a crucial, ubiquitous second messenger that is involved in a myriad of cellular processes. Since astrocytes are considered electrically unexcitable, ${\mathrm{Ca}}^{2+}$ signaling is the main parameter used to monitor their activity in the study of NVC. Thus, not surprisingly, ${\mathrm{Ca}}^{2+}$ signaling in astrocytes has attracted considerable research interest. Understanding astrocytic endfoot ${\mathrm{Ca}}^{2+}$ dynamics, in particular, is key to validating the relationship between astrocytes and the vasculature in NVC, given that the vast majority of the microcirculation in the brain is ensheathed by this specialized subcellular astrocytic compartment. Essential to achieving this understanding is monitoring astrocytic ${\mathrm{Ca}}^{2+}$ signals using appropriate ${\mathrm{Ca}}^{2+}$ sensors and optical tools specifically suited to the task, while simultaneously monitor vascular responses.

Since the first report of a synthetic ${\mathrm{Ca}}^{2+}$ indicator by Tsien^57^ in 1980, a series of ${\mathrm{Ca}}^{2+}$-binding, fluorescent chemical probes with improved optical properties and increased selectivity and affinity have been developed (for review see Paredes et al.^58^). Because ${\mathrm{Ca}}^{2+}$ indicators bind free ${\mathrm{Ca}}^{2+}$, they act as ${\mathrm{Ca}}^{2+}$ buffers; thus, they need to be used at concentrations that do not exceed the buffering capacity of the cells or the organelles under study.^59^ Using indicators with lower ${\mathrm{Ca}}^{2+}$ affinity helps reduce the impact of indicator buffering, but this comes at the cost of reducing signal strength. Engineered acetoxymethyl (AM) esters forms of ${\mathrm{Ca}}^{2+}$-indicator dyes are membrane-permeable, allowing synthetic ${\mathrm{Ca}}^{2+}$ indicator derivatives to be bulk loaded into cells (e.g., astrocytes) for *ex vivo* or *in vivo*. Intracellular ${\mathrm{Ca}}^{2+}$ signaling was first probed in the early 1990s in cultured hippocampal astrocytes using the ${\mathrm{Ca}}^{2+}$-binding fluorescent dye, Fluo-3,^60^ and subsequently in hippocampal brain slices through iontophoretic loading of Calcium Orange.^61^ Astrocytic intracellular ${\mathrm{Ca}}^{2+}$ has since been recorded in anesthetized rats using Fluo-4^62^ and in awake mice with Calcium Green-1-AM.^59^^,^^63^ A common ${\mathrm{Ca}}^{2+}$-indicator dye used in this manner to study astrocytes is Rhod2-AM, reflecting its preferential uptake by astrocytes.^64^ Fluo-4 and Oregon Green have also been used; however, because Fluo-4 and Oregon Green load both neurons and astrocytes, another astrocyte-specific morphological dye, sulforhodamine 101,^65^ must be used to confirm the specificity of these ${\mathrm{Ca}}^{2+}$-indicator dyes in astrocytes.

The first direct evidence in support of a role for astrocytic ${\mathrm{Ca}}^{2+}$ elevations and functional hyperemia in mediating responses to neuronal afferent stimulation was obtained by Zonta et al., who used Indo-1 to measure neural ${\mathrm{Ca}}^{2+}$ oscillations in rat brain slices.^29^ Subsequent works in brain slices using a ${\mathrm{Ca}}^{2+}$-uncaging strategy and the ${\mathrm{Ca}}^{2+}$ indicators Rhod2-AM^48^ or Fluo-4^49^ further suggested that a rise in astrocyte ${\mathrm{Ca}}^{2+}$ is responsible for vascular changes. *In vivo* studies in which astrocytic ${\mathrm{Ca}}^{2+}$ signals recorded using the synthetic ${\mathrm{Ca}}^{2+}$ indicators OGB-1-AM, Rhod-2-AM, or Fluo-4-AM were monitored simultaneously with vascular responses have produced disparate findings, with some studies reporting a rapid onset of astrocytic ${\mathrm{Ca}}^{2+}$ transients after sensory stimulation,^8^^,^^51^ and others reporting astrocytic ${\mathrm{Ca}}^{2+}$ elevations in different astrocytic cellular compartments that are sporadic^9^ and/or delayed relative to functional hyperemic responses.^6^^,^^9^

Although synthetic ${\mathrm{Ca}}^{2+}$ indicators offer a number of advantages, including a broad range of ${\mathrm{Ca}}^{2+}$ affinities and fluorescence spectra, commercial availability, and ease of loading using well-established protocols, there are limitations to their use. These include (1) difficulties in localizing these indicators to a specific cell or particular organelle,^66^ (2) the tendency of synthetic indicators to compartmentalize and eventually become extruded from the cell, and (3) failure of bulk-loaded ${\mathrm{Ca}}^{2+}$ indicators to load astrocytic fine processes.^67^ This latter limitation has been addressed using a patch pipette to load dyes,^68^ but fine processes are still not well loaded using this approach, which has the added drawback of introducing high concentrations of the indicator dye.

**Fig. 1:**
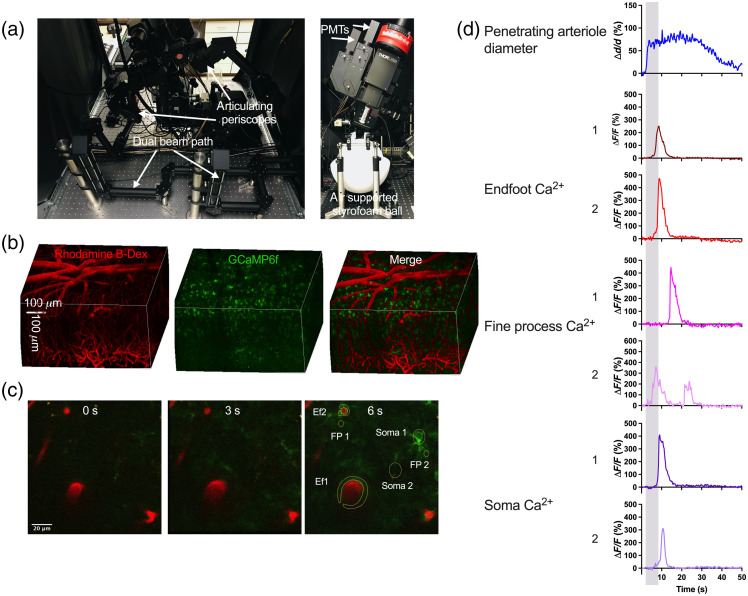
Imaging astrocytic ${\mathrm{Ca}}^{2+}$ and vascular responses to whisker stimulation using a two-photon microscope in a behaving mouse. (a) Layout of a two-photon microscope for awake *in vivo* imaging with dual-beam path and articulating periscopes (left) and air-supported Styrofoam ball for a head-fixed running mouse (right). (b) 3D reconstruction of the barrel cortex of an *Aldh1l1*-Cre-ERT2 GCaMP6f mouse showing astrocytes expressing GCaMP6f (green) and vasculature labeled with Rhodamine B-dextran (red). (c) Arteriole and astrocytic ${\mathrm{Ca}}^{2+}$ responses from different subcellular compartments to 5 s whisker stimulation at different time points. (d) Representative astrocyte ${\mathrm{Ca}}^{2+}$ and arteriole diameter traces from regions of interest shown in Fig. 1(c). Shaded bar indicates whisker stimulation.

The first protein-based ${\mathrm{Ca}}^{2+}$ indicator developed was aequorin, a bioluminescent protein derived from the jellyfish *Aequorea victoria*.^69^ A new GECIs was subsequently introduced by combining the aequorin with green fluorescence protein, yielding GFP-Aequorin.^70^ Exploiting the phenomenon of Förster resonance energy transfer (FRET), researchers developed the Chameleon family of FRET-based GECIs.^71^ Importantly, FRET-based GECIs offer the possibility of ratiometric measurements,^72^ an advantage in *in vivo* studies where it is necessary to resolve motion artifacts or variations in sensor expression among cell populations in a tissue.^72^ Despite these advantages of FRET-based GECIs, a low signal-to-noise ratio, slow kinetics, and large spectral bandwidth^72^^,^^73^ limit their use. In an effort to optimize ${\mathrm{Ca}}^{2+}$ affinity, dynamic range, response speed, and fluorescence properties for imaging at longer wavelengths, researchers have developed a variety of single-wavelength GECIs. These ${\mathrm{Ca}}^{2+}$ sensors, which are typically fusion proteins comprising a fluorescent protein (often circularly permuted) together with the ${\mathrm{Ca}}^{2+}$-binding protein calmodulin (CaM), include camgaroos,^74^ pericams,^75^ GECOs,^76^ and CatchERs,^73^ and GCaMPs.^77^ The most commonly used GECIs for studying ${\mathrm{Ca}}^{2+}$ dynamics are members of the GCaMPs series, from GCaMP2 to various forms of GCaMP5, and GCaMP6^77^^–^^79^ that can be targeted to specific cell types for noninvasive acute and chronic imaging using the Cre-loxP system.^80^ Although new forms, jGCaMP7 and jGCaMP8 have been developed and shown to have improved sensitivity and kinetics, to our knowledge, they have only been tested in neurons^81^ and vascular cells.^80^ The majority of published studies on astrocytes have used GCaMP6 sensors (Fig. 1). It has been shown that GCaMP6s, and GCaMP6m, with slow and medium kinetics, respectively, are more sensitive than OGB-1; GCaMP6s in particular are capable of near 100% detection of action potential in pyramidal neurons, a feat that has not been accomplished previously with protein sensors.^82^ GCaMP6f shows lower detection efficiency but faster kinetics than GCaMP6s and GCaMP6m.^82^ Discordant reports of astrocyte ${\mathrm{Ca}}^{2+}$ responses to stimulation *in vivo* could partly reflect the contribution of different ${\mathrm{Ca}}^{2+}$ sources (i.e., intracellular versus extracellular) or different subcellular compartments to observed ${\mathrm{Ca}}^{2+}$ signals. As such, there is a need for specific ${\mathrm{Ca}}^{2+}$ signal detections not only in organelles but also at locations near the plasma membrane. Khakh and colleagues have further engineered a Cre-dependent, membrane-tethered form of GCaMP6 (Lck-GCaMP6f)^79^^,^^83^^,^^84^ that has faster kinetics^85^ and permits better detection of microdomain ${\mathrm{Ca}}^{2+}$ signals in subcellular structures, such as fine processes that are in close proximity to active synapses.^53^^,^^85^

The powerful impact of GECIs on the study of ${\mathrm{Ca}}^{2+}$ dynamics would not have been fully realized without the development of the Cre-lox/P system. In fact, without appropriate Cre-dependent mouse lines or viruses, studying cell-specific ${\mathrm{Ca}}^{2+}$ dynamics would be neither efficient nor specific. (For details of available Cre-dependent mouse lines for vascular cells and neural cells, please see these excellent reviews.^4^^,^^86^) A number of Cre-dependent transgenic mouse lines (some tetracycline-controllable) that specifically target astrocytes, including those in which Cre expression is driven by *Glt1*, *Gfap*, *Glast*, *Aldh1l1*, and*S100β* promoters, have been developed and optimized. For induction in adult mice, the tetracycline-inducible *Aldh1l1*-Cre/ERT2 mouse line is currently the best mouse line option, offering better temporal control of gene expression, high astrocyte specificity, and efficiency.^85^^,^^87^ The fact that Lck-GCaMP6f can detect more ${\mathrm{Ca}}^{2+}$ microdomains than cyto-GCaMP6f underscores the utility of *Aldh1l1*-Cre/ERT2 in combination with cyto-GCaMP6f or Lck-GCaMP6f, whether delivered via viral vectors (e.g., adeno-associated virus) or transgenic mouse lines, for studying distinct aspects of ${\mathrm{Ca}}^{2+}$ signaling. For example, the use of membrane-tethered forms of GECI has revealed details of localized ${\mathrm{Ca}}^{2+}$ microdomains in endfeet, as well as branchlets.^53^^,^^88^ Notably, the ${\mathrm{Ca}}^{2+}$ fluctuations observed in these processes were preserved in $Ip_{3}r^{-/-}$ mice, an observation that was missed previously using an imaging approach that detected global ${\mathrm{Ca}}^{2+}$ signals in somata.^89^ These distinct ${\mathrm{Ca}}^{2+}$ dynamics in subcellular compartments of the astrocyte have begun to shed light on the sources of astrocyte ${\mathrm{Ca}}^{2+}$. By taking advantage of this ability to detect subcellular sources of ${\mathrm{Ca}}^{2+}$, future studies of NVC employing simultaneous monitoring of ${\mathrm{Ca}}^{2+}$ and functional hyperemia should be able to disentangle the purported integral relationship between astrocytic ${\mathrm{Ca}}^{2+}$ and vascular responses.

## 4 Optical Imaging Tools for *In Vivo* Studies

Imaging techniques such as intrinsic signal optical imaging (ISOI), laser speckle imaging,^90^ laser Doppler imaging,^12^ and blood-oxygenation level-dependent functional magnetic resonance imaging (BOLD fMRI)^91^ provide information about changes in blood flow and/or blood oxygenation, but they are either incapable of high-resolution monitoring of blood flow below the cortical surface (i.e., ISOI) or suffer from low temporal and spatial resolution (e.g., BOLD fMRI). Furthermore, none of these techniques allows simultaneous imaging of blood flow and cellular activity, which is critical for understanding NVC. Confocal laser scanning microscopy allows concurrent measurement of vascular diameter changes and neural ${\mathrm{Ca}}^{2+}$ signals with high resolution; however, deflection and light scattering limit imaging to about 100  μm below the brain surface. Furthermore, the light used for excitation generates fluorescence throughout the entire depth of the sample, making it vulnerable to damage and photobleaching.

In parallel with the development of GECIs, the introduction of TPLSM^92^ provided critical improvements in the ability to image tissues at greater depth with less phototoxicity by virtue of the use of longer excitation wavelengths and because the absorption of light used to excite fluorescent molecules is largely restricted to the focal point.^93^ TPLSM allows *in vivo* imaging of vascular responses concomitantly with neural ${\mathrm{Ca}}^{2+}$ dynamics through an acute or chronic cranial window over the brain region of interest in head-fixed animals.^63^^,^^94^^–^^97^ Fluorescent dyes conjugated to a high-molecular-weight dextran (e.g., FITC-dextran), added to mitigate leakage into brain tissue, can be injected through the tail vein or retro-orbital route to label the vasculature. Spontaneous or activity-dependent vascular responses can be monitored by measuring changes in arteriole diameter and/or red blood cell (RBCs) velocity and/or flux through capillaries, while neural activity can be measured using ${\mathrm{Ca}}^{2+}$ indicators.^6^^–^^9^^,^^51^^,^^54^^,^^98^^–^^100^

TPLSM technology is rapidly developing, with new TPLSM modules offering a number of advanced features for the study of NVC (see Shih et al.^101^ for a detailed discussion of the utility of TPLSM in studying NVC). Utilizing these new features often requires that individual laboratories adapt their existing microscope to the new technologies. However, the cost of even a conventional two-photon microscope is still a limiting factor for many individual laboratories. In many cases, the solution has been custom-built TPLSMs, which can be assembled using off-the-shelf parts at less cost compared with commercial microscopes.^92^^,^^102^^–^^106^ Custom-built two-photon microscopes can also be modular, allowing investigators to adapt to rapidly changing technologies as well as the fast-moving NVC field. Where economics is not limiting, researchers have their choice of a number of commercially available TPLSMs that offer a series of advanced features. Most commercial systems offer high-speed imaging capability using a resonant scan path with a speed up to 30 Hz (512×512  pixels). The tradeoff for faster imaging is typically reduced resolution. Some systems provide rotating body or tilting objectives, allowing different sections of the brain to be imaged without adjusting the position of the animals. Several systems come installed with dual Galvo–Resonant and Galvo–Galvo scanners (Fig. 1), enabling the simultaneous imaging and photoactivation necessary for optogenetics experiments. Tunable lasers are critical for TPLSMs, and the selection of the laser used depends on a number of parameters, including wavelength, laser power, and pulse duration. The shorter the duration of the laser pulse, the more efficient it is at producing a fluorescent signal.^107^ One option with the Ti:sapphire laser is a dual output, in which the laser produces synchronized pulses of tunable laser light and a fixed-wavelength light from an independent output (e.g., InSight X3+). This option eliminates the need for two lasers and is thus advantageous for studies where simultaneous imaging and photostimulation are required.

Despite its advantages over confocal microscopy and ability to simultaneously image vascular responses and cellular activity with low phototoxicity, conventional TPLSM faces challenges in imaging both the vasculature and neuronal populations in deeper brain tissue. A standard TPLSM set-up using commercially available dyes can image the vasculature to a maximum depth of ∼250  μm using the reinforced thinned-skull window preparation (PoRTS)^94^ and ∼900  μm (at high power, 200 mW)^102^ with the open-skull window model, in which both skull and dura are removed. The depth limit for imaging GCaMP6 is ∼450  μm.^108^ A small number of laboratories have used dyes such as Alexa 680-dextran with longer excitation wavelengths (1280 nm) and far-red emission to image the entire depth of an adult mouse cortex (i.e., 1 mm).^109^ Similarly, red-shifted ${\mathrm{Ca}}^{2+}$ indicators (e.g., Cal-590-AM) have been used for imaging neurons through all six layers of the cortex at depths of up to 900  μm.^110^ These developments have begun to address the acute need for deeper imaging *in vivo*, which is required to gain insights into interactions among components of the NVU at different cortical depths. Emerging evidence suggests that sensory-induced functional hyperemia is initiated in deeper cortical layers^111^ and is subsequently conducted upstream to elicit arteriole dilation^112^ is an important driver of efforts to image at greater depths. Recent *in vivo* studies have revealed the heterogeneous nature of astrocytic ${\mathrm{Ca}}^{2+}$ dynamics, demonstrating that different subcellular compartments of the astrocyte display distinct signaling characteristics.^113^^,^^114^ Direct links between discrete ${\mathrm{Ca}}^{2+}$ signals and functional hyperemic responses remain difficult to establish, but future studies that simultaneously examine astrocytic ${\mathrm{Ca}}^{2+}$ responses and vasomotor responses in completely awake animals will help to better elucidate this interaction. Given questions about whether astrocytic ${\mathrm{Ca}}^{2+}$ elevations differently affect functional hyperemic responses observed at arterioles versus those at capillaries,^54^^,^^56^ it will also be critical to examine NVC at different vessel types (i.e., arterioles versus capillaries) as well as at different cortical depths. Imaging in head-fixed, awake animals using two-photon microscopy has revealed behavioral influences on neurovascular activity,^6^^,^^115^ creating the need to image deeper,^6^^,^^112^ subcortical brain regions to investigate the contributions of other neuromodulators, such as serotonin, noradrenaline, and acetylcholine, to NVC in freely-moving animals.

The development of the gradient index (GRIN) lens allows optical access to subcortical and deep brain regions and enables *in vivo* imaging in freely moving animals,^116^^–^^118^ something that is impossible with a conventional lens. Used together with recently developed miniature microscopes with epifluorescent^116^^,^^119^ or two-photon illumination,^120^ the GRINS lens helps realize the possibility of imaging cellular activity concurrently with behavior in freely moving animals. There have been several technological developments since the introduction of a fully integrated, miniature, wide-field, single-photon fluorescence microscope with a light headpiece comprising a light-emitting diode, a microoptic set, and a metal-oxide-semiconductor (CMOS) sensor by Ghosh and colleagues.^119^ These developments include an increased field of view ($7.8\times 4\;{\mathrm{mm}}^{2}$), which comes at the cost of reduced lateral resolution (14  μm),^121^ as well as multicontrast microscopy with a fluorescence channel, an intrinsic optical signal channel, and a laser speckle contrast channel.^122^ A wireless miniature microscope, which eliminates wire entanglement problems associated with optical fibers or electrical wires used by these models to transmit signals, has also been reported.^123^ Although these miniature single-photon microscopes offer some benefits in imaging brain cellular activity in freely moving mice or rats, they all suffer from the lack of optical sectioning capability, low signal-to-noise ratio, and limited tissue penetration (i.e., maximal depth<100  μm). The first miniature two-photon microscope was developed in 2001 used a single-mode optical fiber to transmit near-infrared excitation light, vibrations of the fiber tip for scanning, and a small photomultiplier tube for detection.^120^ The authors were able to image the fluorescently labeled vasculature and ${\mathrm{Ca}}^{2+}$ signals from layer 2/3 pyramidal neurons using Calcium Green-1. Several laboratories have attempted to improve the laser transmission, scanning and detection to image commonly used GECIs in 3D,^124^^,^^125^ but some of these developments have not yet been used in neuroscience.^126^ Most miniaturized microscopes are capable of monitoring either neural activity or hemodynamic activity but not both. To truly characterize NVC, it is crucial that both neural activity and vascular responses be concomitantly examined in freely behaving animals. Senarathna et al. developed a miniaturized microscope that integrates fluorescence, intrinsic optical signals, and laser speckle contrast, and showed that it is capable of imaging neural activity and hemodynamics simultaneously—significant improvement in studying NVC in freely behaving animals.^122^ Although these have not been widely used to study NVC, with continued development, more affordable and flexible miniatured microscopes will become increasingly attractive for use in different research applications, including studying NVC.

**Fig. 2:**
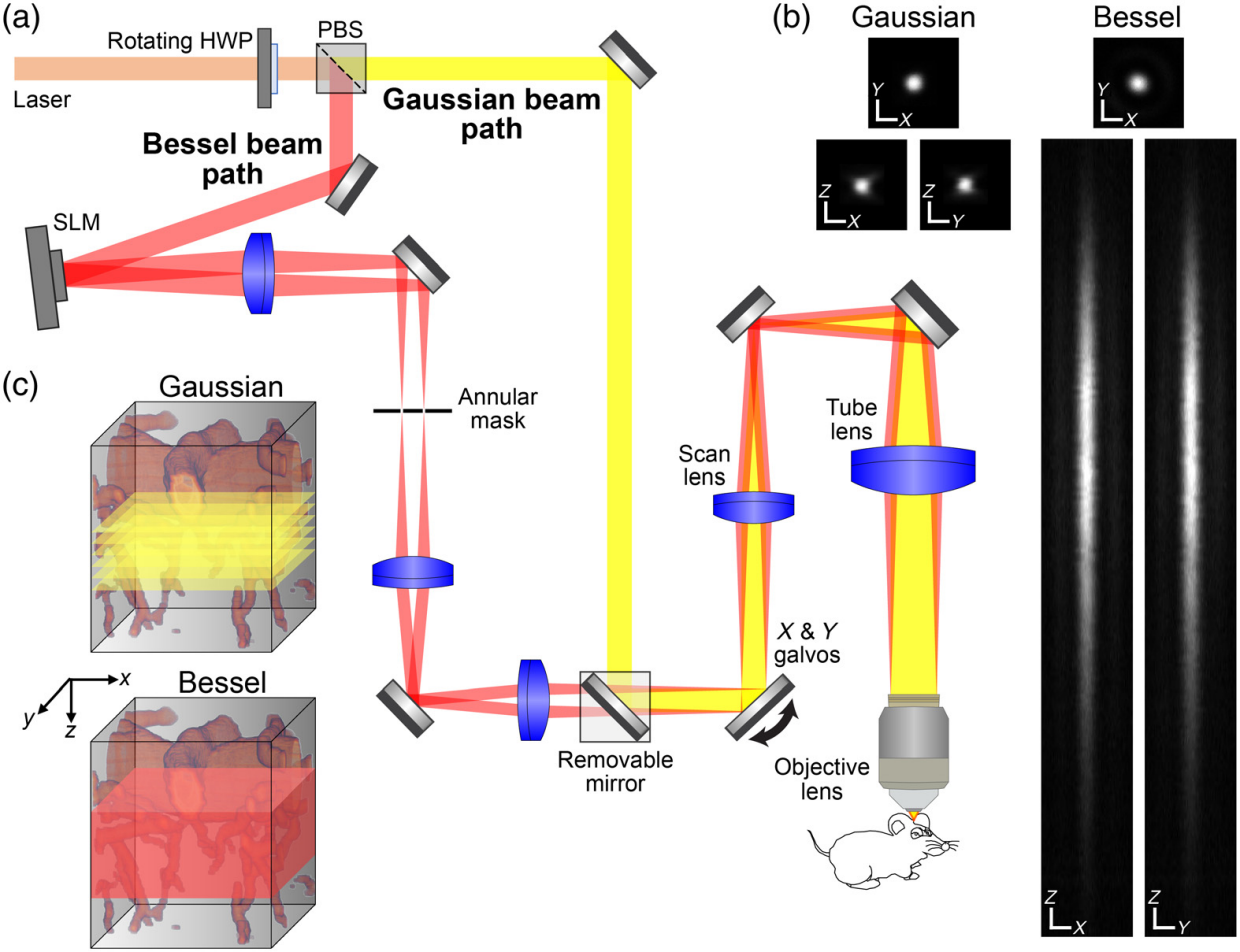
Design and characterization of a commercial two-photon laser scanning microscope with a Bessel focus module. (a) Schematic of the microscope. A half-wave plate (HWP), a polarizing beamsplitter (PBS), and a removable mirror allow switching between Bessel (red) and Gaussian (yellow) beam paths. In the Bessel path, a spatial light modulator and a lens generate an annular illumination pattern, which after spatial filtering by an annular mask is imaged via a 4f system onto the galvos and subsequently imaged via a scan and tube lens pair onto the objective lens back focal plane. (b) Lateral and axial point spread functions for Gaussian and Bessel foci. X and Y scale bars: 1  μm. Z scale bar: 5  μm. Results from one 0.2-μm-diameter bead. (c) Schematic comparison of Gaussian and Bessel volumetric TPLSM methods. Gaussian volumetric imaging requires multiple 2D frames taken at different Z-positions, while Bessel volumetric imaging is achieved with a single frame (reused with permission^130^).

The recently reported heterogeneous nature of astrocytic ${\mathrm{Ca}}^{2+}$ dynamics^114^^,^^127^ and the fact that an individual astrocyte can make as many as 140,000 contacts with neuronal synapses^128^ argue for the development of imaging techniques that allow both deeper and more volumetric imaging. Even though, the intrinsic optical-sectioning feature of TPLSM improves axial resolution and reduces phototoxicity, conventional TPLSM has some limitations in temporal resolution with respect to imaging samples with an extended volume. Moreover, standard TPLSM can monitor vascular diameter changes or blood flow in long segments of horizontally oriented pial vessels spanning the field of view but can only capture a thin cross-section of penetrating arterioles (PAs) and some capillaries, limiting the ability to monitor long PA segments. If conventional TPLSM is used for 3D imaging, the objective needs to be mechanically moved up and down to different depths creating the need to integrate multiple frames and slowing down the acquisition rate. Lu and colleagues have developed an approach for performing rapidly *in vivo* volumetric imaging to a depth of ∼600  μm into the cortex by incorporating a Bessel beam module into a two-photon fluorescence mesoscope.^129^ A Bessel beam is a nondiffracting beam created using an axicon (i.e., conical lens) and a regular lens to extend the depth of view and generate an annular pattern at the back focal plane of the illumination objective lens. In Bessel beam scanning, the focus is laterally confined but axially extended allowing high resolution laterally with a volumetric imaging rate.^129^ The incorporation of Bessel beam focus and a conventional TPLSM with fast volumetric imaging and high resolution makes this a powerful tool for studying neuronal circuitry and, ultimately, for interrogating the integration of neurons, astrocytes, and vascular cells across brain areas and cortical depths in studies of NVC. Although a TPLSM system equipped with a Bessel focus module is commercially available, its utility for studying the true context of NVC is still limited. Recent studies have shown that TPLSM equipped with a Bessel focus module can image cortical vasculature up to ∼600  μm below the brain surface at high speed (up to 99 Hz), allowing 3D monitoring of blood flow from capillaries to upstream pial vessels.^130^ See Fig. 2 for a schematic of the microscope.^130^ The authors further found that the fluorescence brightness of Bessel images changed in proportion to vessel size, providing a simple method for detecting changes in the diameter of blood vessels over an entire volume.^130^ A Bessel beam can also mitigate axial motion artifacts, a common problem experienced in imaging awake behaving animals using a Gaussian beam—and one without an effective corrective measure.

Volumetric imaging with a Bessel beam makes it possible to simultaneously image ${\mathrm{Ca}}^{2+}$ transients from a larger number of low-density neuronal ensembles, such as GABAergic neurons, a functionality that a typical 2D imaging system has a limited capacity to reproduce.^131^ Inhibitory neurons have been implicated in mediating or modulating vascular activity^132^^,^^133^; thus, understanding ${\mathrm{Ca}}^{2+}$ dynamics of these neuronal populations in 3D would help elucidate the interaction. Lu et al.^131^ further found that ${\mathrm{Ca}}^{2+}$ activity was highly synchronized in vasoactive intestinal peptide positive (${\mathrm{VIP}}^{+}$) neurons and was positively correlated with pupil size, an indication of brain state,^134^ whereas ${\mathrm{Ca}}^{2+}$ activity in somatostatin positive (${\mathrm{SOM}}^{+}$) neurons was more heterogeneous and less synchronous.^131^ Interestingly, previous studies have reported that behavioral state affects astrocytic ${\mathrm{Ca}}^{2+}$ dynamics.^6^^,^^115^ The overall picture is slowly becoming clearer, but future studies of the NVU using newly developed, advanced technologies will be needed to bring this picture into focus.

## 5 Future Opportunities

Optical imaging and genetics tools have made significant contributions to research on NVC mechanisms, significantly advancing our understanding of NVC and its essential role in CBF regulation. Much remains to be uncovered; in particular, the underlying mechanisms of NVC and the contributions of neurons and astrocytes to the initiation and maintenance of functional hyperemia remain to be verified. Whether direct targeting of neuronal information to the vasculature takes precedence over the relay of neuronal information from astrocytes to the vasculature, or vice versa, under certain conditions remains an open question. Also unknown is whether both signaling pathways work concomitantly, and if so, under what scenarios might this occur and how might it be advantageous. New evidence continues to provide fresh insights into previously conflicting findings while opening other avenues for examining NVC. Advances in optical imaging in combination with optogenetics and/or pharmacogenetics for monitoring and/or manipulating cellular activity in specific cell types will allow researchers to better interrogate the involvement of individual cells of the NVU in the process. For example, more effort is still needed to elucidate the fascinating and complex variety of astrocytic ${\mathrm{Ca}}^{2+}$ events, particularly discrete signals generated in microdomains, that are observed both spontaneously and in response to increased neuronal activity.^53^^,^^85^ The question remains whether these discrete ${\mathrm{Ca}}^{2+}$ events mediate or modulate functional hyperemia. Recent studies proposed bidirectional communication between arterioles and astrocyte endfeet,^6^^,^^135^^,^^136^ which opens up a new avenue to investigate the astrovascular interaction. Haidey et al. further reported that this communication regulated ultraslow arteriole oscillations.^135^ No single cell or a cell type works in isolation; thus, it is essential to examine the NVU as a whole. Notably, emerging evidence has revealed that animal behavior affects NVC,^6^^,^^115^ highlighting the importance of studying NVC in completely awake animals so as to alleviate the confounding effects of anesthesia. Standard TPLSM provides the basic capabilities for the deep imaging of tissues necessary for the study of NVC. More elaborate systems incorporating better illumination modules for improved resolution and faster scanning rates for volumetric imaging are slowly becoming commercially available. Determining the imaging approach and associated techniques best suited to the question of interest is a critical factor in NVC research. Although newly engineered ${\mathrm{Ca}}^{2+}$ sensors such as GCaMP6s and GCaMP6f have improved sensitivity and faster kinetics, there are still opportunities for future development of red fluorescent ${\mathrm{Ca}}^{2+}$ indicators that would allow deeper imaging in light-scattering tissues. TPLSM with Bessel beam focus enables volumetric imaging but it limits to neural populations of low density. With advances in acquisition-related hardware, the next challenge is developing user-friendly and open source software for data analysis. There are still many missing pieces of the puzzle, and extensive future research will be required to understand the mechanistic basis of NVC and how this process is impaired in disease states. Part of this process will involve a concerted effort to improve imaging tools, available genetic tools, and analytical software for the study of NVC. It can be overwhelming to keep up with rapidly evolving technologies, but the quest remains the same: to better understand NVC.
